# APECED: is this a model for failure of T cell and B cell tolerance?

**DOI:** 10.3389/fimmu.2012.00232

**Published:** 2012-08-02

**Authors:** Nicolas Kluger, Annamari Ranki, Kai Krohn

**Affiliations:** ^1^Department of Dermatology, Allergology and Venereology, Institute of Clinical Medicine, Skin and Allergy Hospital, Helsinki University Central Hospital, University of Helsinki,Helsinki, Finland; ^2^Clinical Research Institute HUCH Ltd,Helsinki, Finland

**Keywords:** AIRE, APECED, endocrine disorders, interleukin 17, interleukin 22, IPEX, T regulatory cells

## Abstract

In APECED, the key abnormality is in the T cell defect that may lead to tissue destruction chiefly in endocrine organs. Besides, APECED is characterized by high-titer antibodies against a wide variety of cytokines that could partly be responsible for the clinical symptoms during APECED, mainly chronic mucocutaneous candidiasis, and linked to antibodies against Th17 cells effector molecules, IL-17 and IL-22. On the other hand, the same antibodies, together with antibodies against type I interferons may prevent the patients from other immunological diseases, such as psoriasis and systemic lupus erythematous. The same effector Th17 cells, present in the lymphocytic infiltrate of target organs of APECED, could be responsible for the tissue destruction. Here again, the antibodies against the corresponding effector molecules, anti-IL-17 and anti-IL-22 could be protective. The occurrence of several effector mechanisms (CD4^+^ Th17 cell and CD8^+^ CTL and the effector cytokines IL-17 and IL-22), and simultaneous existence of regulatory mechanisms (CD4^+^ Treg and antibodies neutralizing the effect of the effector cytokines) may explain the polymorphism of APECED. Almost all the patients develop the characteristic manifestations of the complex, but temporal course and severity of the symptoms vary considerably, even among siblings. The autoantibody profile does not correlate with the clinical picture. One could speculate that a secondary homeostatic balance between the harmful effector mechanisms, and the favorable regulatory mechanisms, finally define both the extent and severity of the clinical condition in the *AIRE* defective individuals. The proposed hypothesis that in APECED, in addition to strong tissue destructive mechanisms, a controlling regulatory mechanism does exist, allow us to conclude that APECED could be treated, and even cured, with immunological manipulation.

## INTRODUCTION

Autoimmune polyendocrinopathy syndrome type 1 (APS-1) or autoimmune polyendocrinopathy–candidiasis–ectodermal dystrophy syndrome (APECED; OMIM 240300) is a rare recessively inherited disorder (Perheentupa, 2002; Betterle and Zanchetta, 2003; Perheentupa, 2006; Husebye et al., 2009). It is caused by mutations in the autoimmune regulator (*AIRE*) gene located on locus 21q22.3 (Bjorses et al., 1996; Nagamine et al., 1997; The Finnish–German APECED Consortium, 1997). APECED displays a worldwide distribution, but specific clusters of high prevalence of the disease are observed among Finns (1:25,000; Ahonen et al., 1990) and Sardinians 1 (1:14,500; Rosatelli et al., 1998; Meloni et al., 2012). It is characterized by the variable association of autoimmune endocrine [hypoparathyroidism (HP), Addison’s disease (AD), hypothyroidism, gonadal insufficiency, insulin-dependent diabetes mellitus, atrophic gastritis, and Biermer’s disease] and non-endocrine disorders (keratitis, malabsorption, vitiligo, and alopecia areata) and a specific predisposition to chronic mucocutaneous candidiasis (CMC). A definite diagnosis of APECED is made upon one of the following criteria: (i) the presence of at least two of three major clinical features: CMC, HP, and AD, or (ii) one disease component if a sibling has already a definite diagnosis, or (iii) disease-causing mutations in both alleles of the *AIRE* gene. However, APECED being highly variable in its presentation, the classical triad may be complete only after years of evolution and diagnose may be therefore missed. Besides, APECED may appear during adolescence or in the young adult (Husebye et al., 2009). Therefore, criteria for a probable APECED have been defined as follows: (i) presence of one of CMC, HP, AD (before 30 years of age) and at least one of the minor components chronic diarrhea, keratitis, periodic rash with fever, severe constipation, autoimmune hepatitis, vitiligo, alopecia, enamel hypoplasia, (ii) any component and anti-interferon antibodies, or (iii) any component and antibodies against NACHT leucine-rich repeat protein 5 (NALP5), AADC, tryptophan hydroxylase (TPH), or TH (Husebye et al., 2009).

## FROM CIRCULATING AUTOIMMUNE ANTIBODIES TO *AIRE, FOXP3*, APECED, AND IPEX

Our knowledge of the nature of the condition now called APS-1 or APECED has increased simultaneously with the general development of immunology and autoimmunity. Since the condition was clearly defined in the end of 1950s and early 1960s, the characteristic clinical picture, the immunological abnormalities and the relationship to other autoimmune endocrine diseases were defined in late 1960s and early 1970s. Furthermore, the genetics of APECED, and the fact that the syndrome was caused by a recessive gene defect – as opposed to the HLA-linked genetics seen in the other solitarily occurring endocrine diseases – were characterized in the 1980s and the target antigens in the organs affected by APECED were molecularly defined in 1990s. A landmark stage in the study of APECED was reached in 1997, when the long sought APECED gene was cloned by two independent groups (Nagamine et al., 1997; The Finnish–German APECED Consortium, 1997). Finally, a new phase in APECED research occurred during the first decennium of 2000, when the autoantibodies toward soluble mediators if immune response were characterized (Meager et al., 2006; Kisand et al., 2011).

The notion that several diseases affecting endocrine organs and earlier defined as idiopathic, were in fact caused by an autoimmune response toward self antigens, became apparent when novel immunological methods became available in 1950s and early 1960s (Blizzard et al., 1963). The association of the three conditions, candidiasis, HP, and AD that were later judged to be the hallmarks of APECED was clearly stated by the groups of Blizzard and Maclaren (Blizzard et al., 1963; Brun, 1978; Neufeld et al., 1981). These groups also defined two clearly distinct syndromes with several associated autoimmune diseases: autoimmune polyglandular syndrome type 1 (PGS-1) and polyglandular syndrome type 2 (PGS-2). The nomenclature was later changed to APS-1 and APS-2, and the former further to APECED (Ahonen et al., 1990; Perheentupa, 2002, 2006; Betterle and Zanchetta, 2003).

Pioneering studies in this field were made especially with the use of immunohistochemistry, demonstrating antibodies reacting with gastric parietal cells in chronic gastritis (Walder et al., 1963; Irvine et al., 1965) and intrinsic factor (IF) in pernicious anemia (Schwartz, 1961; Jeffries et al., 1962), with thyroid epithelial cells in various forms of thyroid diseases (Witebsky et al., 1957; Irvine et al., 1962; Doniach and Roitt, 1964), with the beta cells of Langerhans islands in diabetes mellitus (Kaldany, 1979; Bottazzo et al., 1980), and adrenal cortical cells in AD (Blizzard et al., 1962).

Blizzard’s group noticed that the two polyglandular syndromes, APS-1 and APS-2, differed in their HLA haplotypes (Neufeld et al., 1981). Further studies on the HLA haplotypes revealed that the genetic basis of APS-2, but also of the other isolated forms of endocrine autoimmune diseases found in APS-1, were in the HLA haplotype of the patients. In contrast, APS-1 was shown not to be linked to HLA, and studies with large patient material, collected by Perheentupa’s group in Finland, clearly stated that APS-1 was linked to a recessively inherited gene defect (Ahonen et al., 1990; Perheentupa, 2002, 2006). The autoimmune endocrinopathies could thus be grouped on the basis of their genetic background in two distinct categories: those linked to HLA variation and the one, APS-1 caused by a single mutated gene (**Table 1**). At that stage, however, the responsible gene, the APECED gene, was not yet identified. Once identified, the APECED gene was renamed as *AIRE* in 1997 (Nagamine et al., 1997; The Finnish–German APECED Consortium, 1997).

**Table 1:** Key laboratory findings in the different autoimmune endocrine diseases.

Diagnosis	Clinical findings	Autoantibodies	HLA	Gene defect	Cellular immune response
APECED (APS-1)	Candidiasis and multiple failure of most endocrine organs and non-endocrine autoimmunity	Against all affected organs	No association (?)	Close to 100 mutations described in the *AIRE *gene	CTL against affected organs? Failure inTreg population
APS-2	Addison’s disease with insulin-dependant type I diabetes or thyroid diseases	Against adrenal cortex, pancreatic beta cells, thyroid	Risk haplotypes HLA DR3: DRB1*0301, DQA1*0501,	No single gene defect	CTL against affected organs?
			DQB1*0201		
			DR4		
			DR1, DR7, DR13, and DR14: protective (Betterle and Zanchetta, 2003)		
Addison’s disease	Low levels of gluco- and mineralocorticoid High ACTH, low cortisol, high renin, low aldosterone, subnormal cortisol response to ACTH test: hyponatremia, hyperkalemia	Against P450c21, P450scc	HLA-DRB1-DQA1-DQB1 HLA-DR3	No single gene defect	CTL against affected organs?
IPEX	Enteropathy, diabetes skin disease (mainly eczema), failure to thrive, thyroiditis, recurrent infections	Against enterocytes (autoimmune enteropathy-related 75-kDa antigen) pancreatic-islet cells, insulin, and glutamic acid decarboxilase (GAD), and thyroid (antithyroid microsomal antibodies)	No association	Defective *FOXP3 *gene	Impaired function of regulatory T cells, defective IL-2, IFN-γ, and TNFα- production. Increased production of IL-17

Another immunopathy, termed originally as autoimmune enteropathy (AIE) and later identified as immune dysregulation, polyendocrinopathy, enteropathy and X-linked (IPEX), was described in the 1980s and 1990s (Powell et al., 1982). This disorder was later shown to be caused by a defect in a single gene, *FOXP3* (Bennett et al., 2000). IPEX and APECED are two examples of immune deficiency diseases disclosing both disturbed tolerance and autoimmune phenomena (Moraes-Vasconcelos et al., 2008). Traditionally, reviews tend to associate both IPEX and APECED because of common features. However, both clinical manifestations and predisposition to infections are rather different when comparing both diseases (Moraes-Vasconcelos et al., 2008).

## *AIRE* GENE, MUTATIONS, AND MECHANISM OF ACTION

**FIGURE 1:**
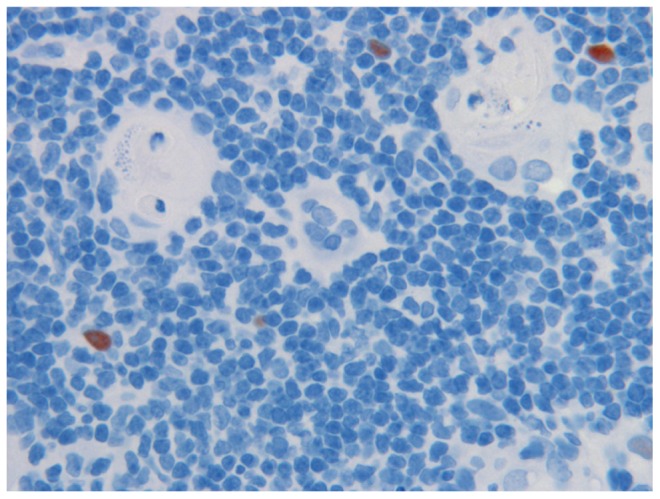
**Medullary epithelia cells in thymus, expressing the AIRE proteins (reddish brown), in close vicinity of the Hassall’s corpuscles (HC) where auto-reactive T cells are thought to be destroyed.** Note cell debris in HC. Magnification 1:40. AIRE was demonstrated with specific monoclonal antibody at 1:2,000 dilution.

*AIRE *is expressed in thymus, lymph nodes, and fetal liver, and encodes a protein with two putative zinc fingers and other motifs suggestive of a transcriptional regulator (Nagamine et al., 1997; The Finnish–German APECED Consortium, 1997). The *AIRE* gene, approximately 13 kb in length, contains 14 exons that encode a polypeptide of 545 amino acids. The AIRE protein functions as a transcription factor (Fierabracci, 2011; Gardner et al., 2009). AIRE is expressed in the thymic medullary epithelial cells (mTECs, **Figure 1**) and in cells of the monocyte/dendritic cell lineage (Kogawa et al., 2002). mTECs through the expression of MHC class II express a wide array of tissue-restricted antigens (TRAs) derived from different organs in the body. TRAs include self-proteins with patterns of expression restricted to a single or small handful of organs. Thymic expression of TRA serves as an important source of self-antigens to allow the negative selection of autoreactive T cells. Collectively, mTEC and thymic monocyte/dendritic cells play a crucial role in establishing self-tolerance by eliminating autoreactive T cells (negative selection) and/or by producing immunoregulatory FOXP3^+^ T cells, which prevent CD4^+^ T cell-mediated organ-specific autoimmune diseases. Collectively, several studies in mouse and man have shown that AIRE regulates thymic expression of several genes of ectopic peripheral proteins including many TRAs. Thus, AIRE dysfunction leads to a decrease in the expression of TRAs in the thymus, and consequently, autoreactive T cell clones escape into the periphery (Derbinski et al., 2005; Moraes-Vasconcelos et al., 2008; Gardner et al., 2009; Fierabracci, 2011)

The most common *AIRE* mutation, the “Finnish mutation,” R257X, affects 82% of Finnish *APECED *alleles (Nagamine et al., 1997; The Finnish–German APECED Consortium, 1997). Interestingly, this mutation occurs also in 70% of the Russian APECED patients studied (Orlova et al., 2010). The same mutation, R257X was also detected in Swiss patients on a different haplotype with closely linked polymorphic markers (Nagamine et al., 1997) and in northern Italian *APECED* patients. Nonsense mutation R139X was found as the predominant haplotype among Sardinian patients (18/20 independent alleles; Rosatelli et al., 1998). Other hotspots have been identified such as the Y85C missense mutation in an isolated Iranian Jewish community (Zlotogora and Shapiro, 1992; Björses et al., 2000). A 13-bp deletion in exon 8 [1085–1097(del)] is ubiquitous and can be found in Norwegians, but also Anglo–Saxons descendant (Zlotogora and Shapiro, 1992) and south Americans (Moraes-Vasconcelos et al., 2008). Today, over 60 different mutations have been described throughout the coding region of AIRE (Akirav et al., 2011).

## CLINICAL PICTURE OF APECED

The clinical picture of APECED is characterized by sequentially occurring diseases, with great variation among the patients as to the severity and time course of the various conditions. In most cases, the affected individual starts suffering from CMC in early infancy or childhood. In most cases, the next organs to be affected are the parathyroid glands, followed by AD and at puberty, hypogonadism mainly in female teens or young adults. Additional clinical features are less common, and include diabetes type I, hypothyroidism, atrophic gastritis with or without pernicious anemia (Biermer’s disease), cutaneous manifestations (alopecia areata, vitiligo, transient skin rash during fever episodes, non-infectious nail dysplasia), ocular symptoms (keratoconjunctivitis, dry eye, iridocyclitis, cataract, retinal detachment, and optic atrophy; Merenmies and Tarkkanen, 2000), enamel dysplasia, hyposplenism/asplenia (implying vaccination against *Streptococcus pneumonia,* *Haemophilus influenzae*, and Hepatitis B as well as antibiotic prophylaxis), autoimmune hepatitis, tubulo-interstitial nephritis, or organized pneumonitis. Involvement of the gastro-intestinal (GI) tract may be responsible for chronic diarrhea, constipation, and malabsorption leading sometimes to malnutrition. GI involvement is difficult to assess as it can be due to numerous various causes that may be associated or follow each other during the life of the patients.

## CANDIDIASIS

Chronic mucocutaneous candidiasis infection by *Candida albicans* is one of the major characteristic of APECED, usually one of first symptoms and most likely the most disabling features of APECED. CMC is naturally not specific for APECED but any child with CMC should be suspected of APECED. According to the Finnish experience, almost all adults with APECED display symptoms of CMC, up to 70% of the patients at the age of 10, up to 94% at age of 20, and 97% at the age of 30 (Perheentupa, 2002, 2006; Betterle and Zanchetta, 2003; Husebye et al., 2009). However, the course and severity vary widely. Oral candidiasis affects the tongue, the buccal mucosa, the gingival, and the pharynx. It ranges in severity from mild form with redness, soreness, angular cheilitis, pseudomembranous lesions, erosions, ulceration and pain to severe chronic inflammation with dysphagia, and development of hyperkeratotic plaques. In the absence of active antimycotic treatment and careful follow-up, chronic oral candidiasis may lead to the development of squamous cell carcinoma with potential lethality by metastatic dissemination. *Candida* esophagitis has been reported to affect 15–22% of the patients (Perheentupa, 2006; Kisand et al., 2011)****with pain while swallowing, retrosternal pain, and dysphagia (Ahonen et al., 1990; Husebye et al., 2009). Chronic esophagitis can lead to local stricture and exceptional esophageal cancer (Rautemaa et al., 2007).

Intestinal candidiasis may cause chronic diarrhea. It should be stressed that esophageal and intestinal candidiasis may occur without any active oral candidiasis. Genital candidiasis affects mainly women with pruritus and vaginal whitish discharge while genital candidiasis seems less frequent in males, possibly underreported due to discrete signs of balanitis. Lastly, *Candida* may affect the nails with chronic paronychia and onychomycosis (Collins et al., 2006). Fingernails are more commonly affected than toenails and the thumbs are the commonest digit affected. This can be explained as infection occurs during the “thumbsucking” period. Management of candidiasis in APECED patients implies an excellent oral hygiene with a careful and regular dental follow-up. Candidiasis should be treated aggressively with antimicrobial therapy and regular prophylaxis should be given.

Any clinically suspicious, chronic thickening or erosion of the mucosa that does not heal should be biopsied to rule out a potential underlying lesion of squamous cell carcinoma. Any difficulties in swallowing or eating or retrosternal pain should prompt to perform esophagoscopy (Rautemaa et al., 2007).

## HYPOPARATHYOIDISM

Hypoparathyroidism is one of the first endocrine features of APECED. Symptoms are related to hypocalcemia, muscle cramps, paresthesia, clumsiness, seizures, and diarrhea. The diagnosis is simply based on blood calcium, phosphorus, and parathormone (PTH) levels: hypocalcemia, hyperphosphatemia, inadapted normal/low PTH without any kidney failure. It is considered that APECED should be systematically considered in cases of primary HP (Husebye et al., 2009). Antibodies against NALP5 as well as against the calcium-sensing receptor of parathyroid epithelial cell have been identified in APECED patients (Gavalas et al., 2007; Kemp et al., 2009, 2010). Patients who are free of HP need an annual monitoring of blood calcium and phosphorus levels. Management of HP relies on daily oral supplementation of vitamin D derivatives and calcium.

## GASTRITIS AND PERNICIOUS ANEMIA

Chronic gastritis, with or without concomitant pernicious anemia belongs to the APECED complex but is found only in a fraction of cases. In non-APECED population, two types of chronic gastritis occur, divided by Strickland into type A and B gastritis. Type A gastritis was known to be caused by autoimmunity while the B gastritis was suspected to be the results of environmental factors. In early 1980s, it was shown by Warren and Marshall (1984) that the major environmental factor was in fact a chronic infection with *Helicobacterium pylori*.

The type A chronic gastritis, with and without pernicious anemia that occur in non-APECED individuals, is clearly linked to certain HLA risk haplotypes, in analogy to isolated AD. In APECED patients, the chronic gastritis differs from the above also in time of occurrence and the speed of the progression. In non-APECED patients, the time needed for progression from the early stage of gastritis, the superficial form to diffuse gastritis, to atrophic gastric and to full gastric atrophy is a slow process, taking up several decennia. Also, the process usually starts in the adult life. In contrast, an APECED-associated gastritic process is much faster and can start in the first decennium of life. Thus, one of the authors of this review was able to follow such a gastric process in two 8-year-old girls with sequential gastric biopsies and could see how, within the time period of only 2 months, the superficial process lead to complete gastric atrophy of the fundus and corpus (K. Krohn, personal experience).

The target molecule for the parietal cell antibodies were shown to be the sodium-potassium channel molecule of the parietal cells on corpus and fundal part of the stomach (Karlsson et al., 1988). In antral gastritis, the antigen are the gastrin-producing cells (Uibo and Krohn, 1984).

Pernicious anemia is the end stage of the gastric immunological destruction, caused partly by the lack of IF, that in addition to the hydrochloric acid is the main product of parietal cells, but also by the autoantibodies recognizing this vitamin B12-binding protein. There are two types of antibodies to IF: one blocking vitamin B12 binding to IF and another type, binding to the IF molecule without interfering with vitamin B12 binding (Toh et al., 1997). Both antibody types prevent the binding of IF to its receptor on the ileal mucosa and subsequent translocation of the vitamin B12 from ileum to circulation.

## ADDISON’S DISEASE

Adrenocortical failure or AD, described by Thomas Addison in the ninetieth century, is considered one of the three hallmarks of APECED, but it occurs also as a solitary disease, or as part of the APS-2 complex. Today, in western word, most cases of AD are caused by autoimmunity, but adrenal cortical destruction and subsequent cortical failure can be caused by several other factors, notably by secondary tuberculosis or other chronic infections. In retrospect, the cases described by Thomas Addison were most likely caused by tuberculosis.

The clinical signs and symptoms of AD are mostly similar in APECED and in solitary AD as well as in APS-2 complex. These include decreased levels of gluco- and mineralocorticoids and elevated ACTH concentrations. The most severe consequence of AD is the life-threatening Addisonian crisis, characterized by general fatigue, dizziness, diarrhea, and death, if the patient is not quickly substituted with corticosteroids, mainly hydrocortisol.

Autoantibodies to adrenal cortex are the characteristic immunological feature in AD, be it part of APECED or APS-2 or the solitary form. These antibodies can be easily demonstrated by immunofluorescence. However, in APECED, but not in the other forms of AD, the autoantibodies are precipitating, and this phenomenon can be demonstrated by Ouchterlony’s immunodiffusion (Andrada et al., 1968; Krohn et al., 1974; Heinonen et al., 1976). In immunodiffusion with APECED serum against adrenal homogenate three precipitating lines were observed, and one of these were shown to represent a mitochondrial antigen while the two others were microsomal.

The nature of the adrenal cortical autoantigens were revealed in early 1990s and shown to be the three main steroidogenic enzymes, P450c17, P450c21, and P450scc (Krohn et al., 1974; Winqvist et al., 1993; Uibo et al., 1994a,b). These three enzymes were also shown to be the ones that could be precipitated by immunodiffusion. The autoantibodies against these three steroidogenic enzymes clearly distinguish the three clinical conditions with adrenal failure: antibodies to all three can be found only in APECED, while in solitary AD and in APS-1, only antibodies to P450c21 are seen. Furthermore, in non-autoimmune AD, caused by tumors or chronic infections, such antibodies do not occur.

## GONADAL FUNCTIONS

Autoimmune oophoritis is responsible for an ovarian insufficiency that may be dramatic for female patients as insufficiency starting in teenagers and young adults. Patients may have either a primary amenorrhea with no or arrested puberty. Other patients develop premature menopause. The diagnosis is confirmed by sexual hormones status; elevated plasma levels of follicle stimulating hormone (FSH) and luteinizing hormone (LH) and low estrogen levels. Autoantibodies against side-chain cleavage enzyme have been related to ovarian insufficiency (Soderbergh et al., 2004) and also steroidogenic enzymes antibodies against cytochrome p450 21-hydroxylase (CYP21A2), cytochrome p450 17α-hydroxylase (CYP17), and cytochrome p450 side-chain cleavage enzyme (CYP11A1).

In female patients, hormonal substitution by estrogen needs to be initiated during puberty. It is strongly advised not to delay pregnancy. In case of hypogonadism, embryo donation has been tried with success.

In males, testicular failure is less common and occurs later. The prevalence of hypogonadism in males is three times lower (8–28%) than in females (35–70%; Perheentupa, 2006). It leads to clinical hypogonadism or isolated azoospermia (Husebye et al., 2009). It has been hypothesized that the blood–testis barrier protects the Leydig cells from an autoimmune attack. However, the physiopathogenic link between circulating autoantibodies and hypogonadism is far from being clear. The two steroidogenic enzymes, p450scc and p450c17, are the main antigens in gonadal failure linked to APECED, but other potential antigenic targets have been identified such as testis-expressed protein TSGA10 (Reimand et al., 2008). However, despite autoantibodies directed against TSGA10 in 7% of the APECED patients, no correlation could be found with gonadal failure (Reimand et al., 2008). One should not forget that the origin of gonadal dysfunction may be related to an authentic-specific autoimmune attack but also be related to other hormonal dysfunction such as AD, pituitary insufficiency, dysthyroidism, or diabetes for instance. Besides, Schaller et al. (2008) suggested that lack of AIRE might affect fertility by disrupting scheduled apoptosis of testicular germ cells. In this respect, the recent hypothesis presented by Matsumoto (2011) that the function of AIRE in thymus would not be in the regulation of transcription but rather in the development and differentiation of the medullary epithelial cells is of primary interest

## OTHER ENDOCRINE DISORDERS

Various other endocrine disorders have been described such diabetes type I mellitus, hypothyroidism, and pituitary failure, the latter diagnosed by a growth hormone deficiency. The diagnosis and management of these conditions does not differ from the standard guidelines for each disorder separately (Perheentupa, 2002; Betterle and Zanchetta, 2003; Husebye et al., 2009).

## OTHER NON-ENDOCRINE DISORDERS

Enamel hypoplasia affect mainly permanent teeth (Perheentupa, 2006), but also deciduous teeth (Pavlic and Waltimo-Sirén, 2009). Pavlic and Waltimo-Sirén (2009) recently suggested that an inadequate process of enamel formation might affect all ameloblasts in phase. Ameloblasts have an epithelial origin with parenchymal cells of endocrine origin. It is speculated that ameloblasts or secreted protein in the extracellular matrix may be the target of autoantibodies leading to hypoplasia. Thereby, APECED would be the first model of dental hard tissue autoimmune disease (Pavlic and Waltimo-Sirén, 2009).

Ocular manifestations affect 25% of the patients and include mainly keratitis that can lead to blindness. It is assumed that the origin of keratitis is the result of autoimmunity against corneal epithelium (Merenmies and Tarkkanen, 2000; Perheentupa, 2006). However, to our knowledge no specific antibodies have been identified in APECED patients. Only antibodies against OBP1 have been found in the AIRE mouse model against lacrimal glands (DeVoss et al., 2010).

Hyposplenism or asplenia is often diagnosed upon the development of thrombocytosis, circulating Howell–Jolly bodies, abdominal ultrasound imaging or in case of severe *S. pneumoniae* infection (Pollak et al., 2009). Destruction of the spleen in APECED has been related to an autoimmune attack against the spleen (Perheentupa, 2002, 2006; Betterle and Zanchetta, 2003; Husebye et al., 2009) although the exact mechanism remains obscure. Again, the mechanisms proposed by Schaller et al. (2008) and by Matsumoto (2011) are of interest, as AIRE expression has also been described in lymphoid tissue and skin. A speculative hypothesis to the evolution of splenic atrophy could thus be disturbance of differentiation, due to lack of AIRE expression.

Various types of GI manifestations are common in APECED patients. These include chronic diarrhea that can be related to HP, severe constipation. Intestinal infection by candida and giardia especially, pancreatic insufficiency and autoimmune enteropathy. Several autoreactive circulating antibodies directed toward intestinal components have been described. Ekwall et al. (1998) identified TPH as an intestinal autoantigen in APECED patients. TPH is expressed in serotonin-producing cells in the central nervous system and in the intestine. In their series of 80 patients, they were able to relate “GI symptoms” to the presence of circulating TPH antibodies and also to the total absence of enterochromaffin cells in the mucosa of small bowel. These enteroendocrine cells (EECs) are scattered through the intestinal mucosa, from the gastric body and antrum to the rectum. They play a key role in growth of the gut, blood flow, motility, secretion of pancreatic enzymes, bile, and bicarbonate-rich fluid (Posovszky et al., 2012). TPH antibodies were found in 89% of the APECED patients with GI symptoms and in 34% of those without (Ekwall et al., 1998). Antibodies can precede clinical symptoms (Ekwall et al., 1998). Conversely, TPH autoantibodies are absent in other inflammatory or autoimmune intestinal diseases. Additionally, Sköldberg et al, (2003) identified also autoantibodies against histidine decarboxylase expressed by EEC – like cells in the gastric mucosa. It is noteworthy, that it is not a routine procedure to perform EECs staining on intestinal biopsies in case of diarrhea or malabsorption, as stressed by Ohsie et al. (2009).****Besides, several studies showed repeatedly that EECs were lacking in the intestinal mucosa and were related to chronic diarrhea (Padeh et al., 1997; Ward et al., 1999; Oliva-Hemker et al., 2006; Posovszky et al., 2012).

Tubulo-interstitial nephritis, life-threatening autoimmune bronchiolitis and other rare manifestations have also been reported in APECED (Perheentupa, 2002, 2006; Betterle and Zanchetta, 2003; Husebye et al., 2009). The main identified autoantibodies are summarized in **Table 2**.

**Table 2:** Main identified target of autoimmune antibodies inAPECED patients.

Diagnosis	Main identified circulating autoantibodies
Addison’s disease	21 hydroxylase, 17α hydroxylase Side-chain cleavage enzyme antibodies (or steroid cell antibodies)
Hypoparathyroidism	NALP5, Ca^2+^ sensing receptor
Hypothyroidism	Thyroperoxydase
	Thyroglobuline
Hypogonadism	17α hydroxylase
	Side-chain cleavage enzyme antibodies (or steroid cell antibodies)
Diabetes type I	Glutamic acid decarboxylase 65-kDa isoform (GAD65)
	Insulin
	Tyrosine phosphatase (IA2)
Pituitary insufficiency	Tudor Domain containing protein 6 (TDRD6)
Atrophic gastritis/	Intrinsic factor, gastric parietal cell
Biermer’s disease	
Intestine	Glutamic acid decarboxylase 65-kDa isoform
	(GAD65)
	Histidine decarboxylase
	Tryptophan hydroxylase
Autoimmune hepatitis	Aromatic L-amino acid decarboxylase (AADC)
	Cytochrome P450 1A2
	Cytochrome P450 2A6
	Cytochrome P450 1A1
	Cytochrome P450 2B6
Vitiligo	Transcription factors: SOX 9, SOX 10, aromatic L-amino acid decarboxylase (AADC)
Alopecia areata	Tyrosine hydroxylase
Nephropathy	Antibody against tubular basement membrane (Hannigan et al., 1996)
Pulmonary disease	Potassium channel regulatory protein (KCNRG)
Eye	OBP1 *
Non-tissue specific**	IFN-α, IFN-β), IFN-ω, IL-22, IL-17F, IL-17A

* Identified in a mouse model AIRE^-/-^.

** Main non-tissue-specific antibodies according to Kisand et al. (2011).

## TREATMENT

Management of APECED relies in education of the patients to know his disease, education of the local physician, and the knowledge that new components of the disease may develop during life. Psychological support is strongly recommended as this disease impairs greatly the quality of life of the patients (Perheentupa, 2006). Except candidiasis treatment that has been explained previously, treatment of APECED relies mostly on hormone replacement therapy according to affected organs (thyroid, parathyroid, pancreas, etc.). In some rare and potentially lethal situations, however, patients may require corticosteroid treatment in association with immunosuppressive therapies. These rare situations include autoimmune hepatitis, especially its fulminant form, which may be lethal and therefore prompt immunosuppressive therapy is needed (Obermayer-Straub et al., 2001). The same is true for interstitial nephritis and bronchiolitis in association to APECED. Immunosuppressive therapies have been also proposed in case of severe intestinal malabsorption with efficacy (Padeh et al., 1997; Ward et al., 1999). Very recently, Rituximab, a chimeric monoclonal antibody targeting B cell lymphocytes expressive CD20 has been successfully used in a young patient with bronchiolitis (Popler et al., 2012). The rationale for Rituximab use in APECED is supported by the presence of B cell infiltrates in the affected organs (Gavanescu et al., 2008).

## AUTOANTIBODIES TOWARD INTERFERONS AND CYTOKINES

At the beginning of this millennium, the antibody responses to the main target organs affected in APECED, and the responsible target antigens were fairly well characterized. A new period in APECED studies started along the publication by Meager et al. (2006), describing high-titer antibodies to several type I interferons in practically all APECED patients studied. This anti-interferon response was exceptionally strong, since serum titers up to 1:1,000,000, and clearly exceeding the titers seen against organ-specific antigens, were found.

Furthermore, high-titer antibodies were seen against the two main mediators secreted by Th17 cells, interleukin-17 and interleukin-22 (IL-17 and IL-22). Responses with lower titers were occasionally seen against other interleukins, too. In our own as yet unpublished observations we have detected occasional high-titer responses against several other interleukins and chemokines, as well, but in contrast to the aforementioned responses, these responses are not characteristic to all APECED patients but rather occur occasionally in only a few patients.

The significance of these novel findings are still unclear, but some information concerning the role of IL-17/IL-22 antibodies in the chronic candida infections, characteristic for APECED, has been obtained. Th17 cells secrete IL-17 and IL-22, which are cytokines with potent antifungal properties (Engelhardt and Grimbacher, 2012) and the occurrence of autoantibodies against IL-17/IL-22 were reported to closely correlate to the presence of candida infection (Kisand et al., 2011; Engelhardt and Grimbacher, 2012). However, recent evidence points to a new interaction between AIRE and dectin-1, a pattern-recognition receptor that is important in antifungal innate immunity. Pedroza et al. (2012) recently showed that AIRE participates in the dectin-1 signaling pathway, and thus, missing AIRE activity could contribute to fungal susceptibility through this pathway. Dectin-1 is expressed on phagocytes and was recently shown to induce a non-canonical caspase-8 inflammasome in response to fungal and mycobacterial infection (Gringhuis et al., 2012). The activation of the dectin signaling pathway also leads to expression of IL-17 and 22 and defensins, however. Besides, other mechanisms such Dominant-negative mutations in STAT3, gain of mutation of STAT1, mutations in IL-17F and IL-17R may be alternate causes of CMC (Engelhardt and Grimbacher, 2012).

The significance of the antibody response toward interferons and other cytokines is presently also unclear. One could speculate that some of these antibodies against type I interferons as well as reacting with IL-17 and IL-22 might have a protective function. As pointed out by Waterfield and Anderson (2011), antibodies to type I interferons do not seem to lead to increased susceptibility to viral infections. This resistance might be due to redundancy and it has to be seen whether this anti-interferon response is directed only toward certain members of the interferon family. While Th17 cell response and the release of soluble IL-17 and IL-22 are evidently necessary for the defense against mucocutaneous candida infection, the same cytokines have a role in the development of psoriasis. Similarly, interferons are known to be involved in the pathogenesis of several conditions, and one such chronic ailment is the autoimmune diseases belonging to the systemic lupus erythematosus (SLE) complex. Anti-interferon alpha antibodies are currently being tested as a therapeutic mean against SLE (Merrill et al., 2011). In order to be able to find out if the antibodies against interferons and other cytokines could have a protective role in APECED, large APECED patient cohorts have to be studied in order to find out whether, e.g., psoriasis and SLE are significantly less common in APECED patients than in the general population.

The reason for the antibody response toward soluble immune mediators is still unclear, and we do not yet know what exactly elicits them and thus, only speculative scenarios can be presented. It is conceivable to hypothesize, however, that the tissue destruction preceding the failure of the endocrine organs may have a role. Tissue destruction, be it caused by trauma, viral infection or autoimmune attack, would probably lead not only to the release of potential tissue-specific autoantigens and thus, to autoantibody formation against these proteins, but could also lead to an inflammatory response and production of several mediators of inflammation. One key group of molecules in this respect is the acute phase proteins, notably those belonging to the IL-1 group.

It is generally believed that the destruction of the endocrine organs in APECED is caused by the autoreactive CD8^+^ cytotoxic T cells, although definitive evidence for this mechanism is still lacking (Betterle and Zanchetta, 2003; Moraes-Vasconcelos et al., 2008). This hypothesis is reinforced by the examination of microscopic examinations of samples, sometimes obtained post-mortem. Indeed, parathyroid, adrenal glands, or ovaries pathology disclosed also atrophy and lymphocytic infiltration that suggest lymphocytic aggression of the organs leading to atrophy and dysfunction (Betterle and Zanchetta, 2003). This is also stressed, indirectly, by the analysis of the AIRE-deficient mouse model, who develop also a lymphocytic infiltration in some inner organs along with atrophy (Ramsey et al., 2002).

However, cell destruction caused by an immune response against the endocrine organ would in fact lead to a similar situation that is thought to happen in viral infections. In fact, several autoimmune diseases, such as diabetes type I or chronic autoimmune liver diseases are thought to be a consequence of preceding viral infection: enterovirus infection in the case of diabetes type I and hepatitis B in the case of chronic active hepatitis. In viral infections, a specific group of intracellular regulatory molecules, TRIMs (tripartite motif-containing proteins), have been shown to have a key role in eliciting an autoimmune or auto inflammatory consequence (Jefferies et al., 2011).

The TRIM protein family is a form of RING domain containing E3 ligases and they exert a variety of biological functions, related to immunity and inflammation (Jefferies et al., 2011). Specifically, of the more than 20 different TRIM proteins, some seem to up-regulate the expression of type I interferons and proinflammatory cytokines, notable interleukin-1beta (IL-1beta). Furthermore, the same mediators of immune response and inflammation are in some cases known to up-regulate the expression of TRIMs. Thus, a vicious circle can theoretically occur and this in turn could lead to autoimmunity. So far, overexpression of TRIMs, or an autoimmune response toward them, has been shown to be linked to autoimmune and autoinflammatory processes in Sjögren’s syndrome or rheumatoid arthritis (Jefferies et al., 2011).

Presently, we have no information how the occurrence of autoantibodies toward the interferons and other mediators of immune response might affect the aforementioned vicious circle, but it is conceivable to speculate that such an antibody response could have an balancing effect. One could thus form a hypothesis, that in APECED, the primary defect outside thymus, where the autoreactive T cells are not destroyed, would be the cell destruction of the endocrine organs by cell-mediated immune response, followed by release of cellular components taken up by professional antigen presenting cells and further stimulating the activation of CD4^+^ Th-cells and finally resulting in an autoantibody response to these organ-specific antigens. However, simultaneous overexpression of TRIMs and subsequent up-regulation of a variety of soluble mediators of immune response and inflammation, such as interferons and members of the IL-1 family would lead to autoantibody formation also against these cytokines. Lastly, one reason for the break of tolerance to immune mediators, and subsequent production of autoantibodies could be related the fact that AIRE expression seems to occur, in addition to thymic epithelial cells also outside thymus, notably in dendritic cells, that normally express also such mediators (Heath and Carbone, 2009)

The consequences of such cytokine-directed antibody response are still an open question. In case of the Th17 type interleukins (IL-17 and IL-22) there is convincing evidence that such antibodies are linked to the CMC. However, at least in some cases, the antibodies may have a balancing, down-regulating effect on the expression of the corresponding biologically active molecules but also, by regulating the immune response to target organs. Thus, it is possible to presume, that especially the antibodies to type I interferons might have a protective effect, as some chronic immune diseases, such as psoriasis and SLE, are rare or non-existing among APECED patients.

## CELL-MEDIATED IMMUNE RESPONSES

Although it is now a generally accepted view that the consequence of the AIRE defect in APECED will lead to the escape of the potentially autoreactive T cells, there is in fact rather little direct evidence to show that the tissue destruction in the endocrine organs affected in APECED is caused by cytotoxic CD8^+^ T cells. Furthermore, most studies describing the phenotype of the lymphocytes infiltrating affected organs is not from APECED patients directly, but from patients suffering of solitary lesions that are similar to the ones seen in APECED, such as solitary AD or diabetes. However, the solitary endocrine diseases, such as isolated thyroid disease or AD are remarkably similar in their clinical picture as well as immunological findings as those of APECED. Thus, in solitary AD and in APECED with adrenocortical failure, autoantibodies recognize the p450c21 steroidogenic enzyme. Interestingly, in this disease complex CD8^+^ T cells that reach against specific T cell epitopes in p450c21 has been demonstrated (Bratland et al., 2009; Rottembourg et al., 2010) Likewise, in thyroid diseases, thyroglobulin and thyroid peroxidase are recognized by the autoantibodies, irrespective if the condition is occurring alone, in association of APS-2 or as part of the APECED complex. The similar synergism in terms of the nature of autoantigens occurs in chronic immunological liver diseases, too.

In chronic aggressive hepatitis the lymphocytic infiltrating cell population has been shown to be of the CD8^+^ lineage (Si et al., 1984). In a murine model of Graves’ disease, the CD8^+^ cell population contains also the recently identified CD8^+^CD122^+^ T cells that are functionally similar to the CD4^+^CD25^+^ regulatory T cells (Ryan et al., 2005). Furthermore, studies in thyroid and other affected organs show that one of the main cell population in the lymphocytic infiltrate are in fact the CD4^+^ Th17 cells that secrete as effector molecules, the cytokines IL-17 and IL-22. In experimental autoimmune diseases, the balance between the Th17 effector cells and the two regulatory T cells, CD8^+^CD122^+^ and CD4^+^CD25^+^, seems to regulate both the occurrence and severity of tissue destruction and functional failure.

There could thus be two distinct mechanisms operating in the pathogenesis of autoimmunity in the endocrinopathies: one mediated by soluble effector molecules, such as IL-17 and IL-22 as well as type I interferons, and an other one mediated by effector T cells, which are either of the CD8^+^ CTL cell or of the Th17 effector cell lineage. To counteract these, again two distinct biological processes would occur: the production of autoantibodies and secondly, the emergency of the regulatory T cells. As to the regulatory T cell response, it is to note that one key immunological failure in APECED, is the dysregulation of the Treg cell maturation (Ryan et al., 2005; Kekäläinen et al., 2007; Saitoh et al., 2007; Laakso et al., 2010, 2011; Wolff et al., 2010).

In normal thymus, Treg maturation follows a preprogrammed scheme, and the immature CD8^+^CD4^+^FOXP3^+^ seems to be prone to apoptosis, whereas the more mature form CD4^+^CD8^-^FOXP3^+^ cells form the active Treg population (Lehtoviita et al., 2009). According to Endharti et al. (2011) the CD8^+^CXCR3^+^ Tregs in humans are functionally similar to murine CD8^+^CD122^+^ Tregs. Furthermore, in APECED patients the recent thymic emigrant (RTE) pool of Treg cells shift to the activated pool and the RTE reservoir is depleted. Most importantly, in APECED patients these cells express less FOXP3 than in the healthy controls (Laakso et al., 2010). Thus, in APECED the newly formed Treg cells have a developmental defect and their function is therefore impaired. Data concerning the CD8^+^ regulatory T cells in APECED patients is missing, however.

The finding that the regulatory T cell population in APECED is functionally defective and that the expression of the key molecule for Treg function, the FOXP3 is impaired, is consistent with clinical findings in IPEX syndrome, caused by a defect in the function of the FOXP3 gene. However, it should be noted that the effect of FOXP3 mutations in Treg population also in the IPEX patients is highly variable. Also, in contrast to APECED there seems to be a genotype–phenotype correlation in IPEX, as different mutations are associated in variable clinical picture, that show differences in severity as well in the types of clinical components that are present (Torgerson et al., 2007; d’Hennezel et al., 2009) A consistent finding in IPEX is however the inability of the CD4^+^CD25 high Tregs to suppress the function of autologous effector T cells (Bacchetta et al., 2006). There are, thus, several differences in the clinical picture of APECED and IPEX, but both conditions show clear immune destruction of at least some endocrine organs. Both conditions also share some similarities in the GI symptoms.

The proposed hypothesis that in APECED both tissue destructive mechanisms and controlling regulatory mechanisms exist raises a question whether APECED could be treated or even cured by immunological manipulations. To find an answer for this question is one of the further challenges for APECED research

## Conflict of Interest Statement

The authors declare that the research was conducted in the absence of any commercial or financial relationships that could be construed as a potential conflict of interest.

## Abbreviations

AADC: aromatic L-amino acid decarboxylase
AD: Addison’s disease;
AE: autoimmune enteropathy
APS-1: autoimmune polyendocrinopathy syndrome type 1
APECED: autoimmune polyendocrinopathy–candidiasis–ectodermal dystrophy;
CMC: chronic mucocutaneous candidiasis
EECs: enteroendocrine cells
GI: gastro-intestinal
HP: hypoparathyroidism
IPEX: immune dysregulation, polyendocrinopathy, enteropathy and X-linked
IF: intrinsic factor
IL-1: interleukin-1
IL-17: interleukin-17
IL-22: interleukin-22
mTECs: thymic medullary epithelial cells
NALP-5: NACHT leucine-rich-repeat protein 5
PE: promiscuous expression
PTH: parathormone
SLE: systemic lupus erythematosus
TH: tyrosine hydroxylase
TPH: tryptophan hydroxylase
TRIMs: tripartite motif-containing proteins
TSA: tissue-specific antigens.
